# Effects of Protease Addition and Replacement of Soybean Meal by Corn Gluten Meal on the Growth of Broilers and on the Environmental Performances of a Broiler Production System in Greece

**DOI:** 10.1371/journal.pone.0169511

**Published:** 2017-01-03

**Authors:** Ilias Giannenas, Eleftherios Bonos, Vasileios Anestis, Georgios Filioussis, Dimitrios K. Papanastasiou, Thomas Bartzanas, Nikolaos Papaioannou, Athina Tzora, Ioannis Skoufos

**Affiliations:** 1 Laboratory of Nutrition, School of Veterinary Medicine, Aristotle University of Thessaloniki, Thessaloniki, Greece; 2 Research Institute of Animal Science, ELGO-Dimitra, Paralimni Giannitsa, Pella, Greece; 3 Laboratory of Agricultural Engineering and Environment, Institute for Research and Technology of Thessaly, Centre for Research and Technology Hellas, Volos, Greece; 4 Laboratory of Agricultural Constructions and Environmental Control, Department of Agriculture Crop Production and Rural Environment, School of Agricultural Sciences, University of Thessaly, Magnisia, Greece; 5 Laboratory of Microbiology and Infectious Diseases, School of Veterinary Medicine, Aristotle University of Thessaloniki, Thessaloniki, Greece; 6 Laboratory of Pathology, School of Veterinary Medicine, Aristotle University of Thessaloniki, Thessaloniki, Greece; 7 Department of Agricultural Technology, Division of Animal Production, Technological Institute of Epirus, Arta, Greece; Leibniz-Institut fur Pflanzengenetik und Kulturpflanzenforschung Gatersleben, GERMANY

## Abstract

An experimental study was conducted to examine the combined effects of adding a dietary protease, reducing the levels of soybean meal (SBM) and introducing corn gluten meal (CGM) in the ration of a group of broilers reared on a commercial Greek farm. Five hundred forty chicks were divided into three dietary treatments with six replicates of thirty birds each. The first group (Control) was fed a conventional diet based on corn and soybean meal, containing 21% w/w crude protein (CP). The second group (Soy-Prot) was supplied a corn and SBM-based diet containing a lower level of CP (20% w/w) and 200 mg of the protease RONOZYME^®^ Proact per kg of feed. The third group (Gluten-Prot) was fed a diet without soybean-related constituents which was based on corn and CGM and with CP and protease contents identical to those of the diet of the Soy-Prot group. Body weight, feed intake, feed conversion ratio (FCR), intestinal microbiota populations and morphology, meat quality and cost were evaluated. Furthermore, a partial life cycle assessment (LCA) was performed in order to assess the potential environmental performance of the systems defined by these three dietary treatments and identify their environmental hot-spots. The growth performance of the broilers supplied the Soy-Prot diet was similar to the broilers supplied the Control diet. However, the broilers which were fed the Gluten-Prot diet at the end of the trial showed a tendency (P≤0.010) for lower weight gain and feed intake compared to those of the Control diet. When compared to the Control group, lower counts of *C*. *perfringens* (P≤0.05) were detected in the ileum and cecum parts, and lower counts of *F*. *necrophorum* (P≤0.001) were detected in the cecum part of the birds from the Gluten-Prot group. The evaluation of intestinal morphometry showed that the villus height and crypt depth values were not significantly different (P>0.05) among the experimental groups for the duodenum, jejunum and ileum parts. No significant differences (P>0.05) were observed in the quality of the breast and thigh meat and in the feed cost per kg body weight gain for the total duration of the growth period between the Control and Gluten-Prot broiler groups. The LCA suggested that the ammonia and nitrous oxide emissions due to litter handling constitute the farm level hot-spots for the Acidification and Eutrophication Potentials of the Control and Soy-Prot systems and the Global Warming Potential of the Gluten-Prot system, respectively. The Latin American soybean production and domestic corn production and lignite mining are important off-farm polluting processes for the studied life cycles. The Soy-Prot and Gluten-Prot systems both performed better than the Control system in nine of Environmental Impact Category Indicators assessed, with the respective differences being generally larger for the Gluten-Prot system. The environmental impact estimates are regarded as initial, indicative figures due to their inherent uncertainty. Overall, the results could be considered as positive indications in the effort to sustainably replace the conventional, soybean-dependent control diet in the specific broiler production system.

## Introduction

The poultry industry in Europe relies heavily on imported proteinaceous feedstuffs and mainly on soybean meal (SBM) [1]. SBM is generally a consistent, high quality product [2], but unfortunately, its price can be prohibitive and many poultry producers are looking for alternative sources of supplementary protein available at a lower cost. Other constrains on the use of SBM in poultry diets are the serious consumer concerns on the environmental impact of soybean production and use, as well as the fact that most of the imported SBM from US and Latin American countries is genetically modified (GMO). For this reason, the investigation for alternative proteinaceous feeds to SBM is still current and important [1].

Corn gluten meal (CGM), is another common protein ingredient found in animal diets. It is a by-product from the manufacturing of corn syrup or starch. CGM is the dried residue after the removal of bran, germ and starch [3]. CGM could be an excellent source of dietary protein, since nearly 600 g/kg of the dry matter is crude protein and the sulphur amino acids are highly digestible by several animal species [4, 5]. Several research papers have been published, describing the digestibility of CGM by non-ruminants. CGM has often been added to broiler chick diets because it is a good source of sulphur amino acids and is highly available [4]. In the study of Knabe *et al*. [6], CGM was found to be the most digestible feed ingredient among the plant proteins supplied to growing pigs. Despite the fact that CGM is not a cheap product due to its higher protein content, it could replace SBM effectively by being included to broiler rations at lower levels.

Public concern about the use of feedstuffs with different origins or enzymes in livestock production has increased in recent years as feed production chain and livestock production have been related to significant environmental impacts [7]. The use of exogenous protease enzymes is considered as a method to reduce the feed’s protein level without inducing negative effects on growth performance. The use of exogenous enzymes to improve the performance of poultry is not a new concept and has been extensively studied and reviewed [8–11]. However, although the efficacy of carbohydrases, proteases, and phytases in the diets of poultry has been well established, there is still a great deal of uncertainty regarding the modes of action of exogenous enzymes. Furthermore, the countless interactions between enzymes and the host animal, its microflora, and also dietary ingredients are not fully understood [10, 12, 13]. Recently, it was shown that a protease in combination with organic acids and plant essential oils can positively affect the performance of chickens [13].

Substitution of SBM in broilers’ diet by various alternative protein sources such as CGM should also be evaluated in terms of both the feed cost and the modification of broilers’ carcass quality. Poultry meat has relatively low and competitive pricing compared to other meat. Also, the absence of cultural or religious obstacles, and dietary and nutritional (protein) qualities are the main factors explaining its attractiveness [14].

Modifications in poultry diets can be further supported in the framework of sustainability of poultry production processes, if their influence on the environmental impacts of these processes is positive. Life Cycle Assessment (LCA) is a methodology that could be used for such evaluations. LCA takes into consideration the entire supply chain of a product and it has been recognised as a valuable method for the estimation of the environmental burden which is connected to livestock production processes [15–17]. The interest of the poultry industry to use LCA for the evaluation of environmental impacts of poultry production processes has also become apparent after the publication of the document “Greenhouse gas emissions and fossil energy use from poultry supply chains: Guidelines for assessment” from the recently established Livestock Environmental Assessment and Performance Partnership (LEAP), under the coordination of FAO [18].

Several authors have attempted to apply the LCA methodology in the broiler supply chain [19–23]. To this day, the effect of reducing the content of soybean meal in broiler diets on the environmental performance of the system has been examined by a limited number of studies [24–26]. The study of Leinonen *et al*.[24] is the only study found in the literature, using LCA to compare the environmental impacts of broiler production systems defined for alternative to soybean protein sources in the broilers’ diet. Moreover, in another study [25], LCA was utilized to investigate the effect of a combination of digestibility-improving enzymes (xylanase, α-amylase and protease) in the diet of broilers, on the carbon footprint of commercial broiler production. Furthermore, in the work of Leinonen and Williams [26], LCA was applied to examine the effect of adding the protease Ronozyme^®^ ProAct in different broiler diets with reduced content of soybean-based products on a number of environmental impact categories for a standard UK broiler production system. These studies underlined the increased scientific and industrial interest to comparatively assess the overall environmental performance of the systems defined for different broiler diets, in an effort to sustainable reduce the dependence of poultry diets on soybean production.

The aim of the present study was to experimentally investigate the combined effects of adding a dietary protease, reducing the levels of soybean meal and introducing CGM in the ration of a group of broilers reared on a commercial Greek farm, on their growth and health performances. A small reduction in the soybean meal level, as well as their total substitution was examined. Moreover, the effect of the tested alternative diets on the feed cost and the quality of produced broiler carcass in terms of its chemical composition was studied. An LCA approach was finally adopted in order to evaluate the potential environmental performance of the broiler production systems corresponding to the alternative experimental diets and identify their environmental hot-spots.

## Materials and Methods

### Ethics statement

This study was carried out in strict accordance with the guidelines of “Council Directive 86/609/EEC regarding the protection of animals used for experimental and other scientific purposes”. The trial protocol was approved by the Institutional Committee for Animal Use and Ethics of The Technological Institute of Epirus, Department of Animal Production (Nr: 11SYN_3_47-01/03/2013). Throughout the trials, the birds were handled in compliance with local laws and regulations and in accordance to the principles and guidelines for poultry welfare [27].

### Protease and chemicals

The protease (RONOZYME^®^Proact (PRA), DSM Nutritional Products, Basel, Switzerland) used in this study is a commercial enzyme produced by submerged fermentation of *Bacillus licheniformis* containing transcribed genes from *Nocardiopsis prasina*. All other chemicals were purchased from Sigma-Aldrich Chemical Co.

### Birds housing and management

A growth experiment was performed in a broiler house of commercial broiler farm in Basilika (Latitude: 40°48΄; Longitude: 23°14΄; Height 69 m), Greece. Rice husk was used as bedding material. The stocking density was 15 birds per m^2^. During the experiments, commercial breeding and management procedures were applied. Mechanical ventilation was applied, the ventilation rate being 0.5–3.0 m^3^/hr in order to control ambient temperature levels with air NH_3_ level lower than 10 ppm. Natural and artificial light was provided on a basis of 23 h for the first 2 days, 16 hours from day 3 to day 14, 21 h from day 15 to the end of the trial. Feed and drinking water were offered to all birds *ad libitum* throughout the experiments.

### Feeding trial, economics and carcass traits

The feeding trials were conducted with 540 one day-old Ross 308 as hatched broiler chicks that were bought from a commercial breeder in Galatista, Greece. The chicks were randomly allocated into three groups with six replicates. Each replication (30 birds) was housed randomly in a different floor pen of the same broiler house. During the feeding period that lasted 42 days, a different diet was supplied to each group (Table 1). The first group (Control) was fed a conventional diet with corn and SBM containing 21% w/w of crude protein (CP), based on NRC [28] and Ross Aviagen recommendations. The second group (Soy-Prot) was supplied a corn and SBM-based diet containing a lower level of CP (20% w/w) and 200 mg protease per kg of feed. The third group (Gluten-Prot) was fed a diet without soybean-related constituents which was based on corn and CGM and with CP and protease contents identical to those of the diet of the Soy-Prot group. One issue that remains when the SBM is totally replaced is the balance of amino acids, protein and energy in the diet. In this study, we used supplementary quantities of commercial amino acids and different quantities of raw materials in order to equalize diets in all experimental groups.

**Table 1:** Ingredients and chemical composition of diets.

Ingredients, g/kg	Group		
	Control	Soy-Prot	Gluten-Prot
Corn	597.0	630.4	650.4
Soybean meal, 47% CP	338.0	307.3	-
Corn gluten meal	-	-	201.0
Wheat bran	-	-	100.0
Soybean oil	30.4	25.3	-
Limestone	17.4	17.4	22.5
Dicalcium phosphate	7.3	7.8	8.1
Sodium chloride	2.7	2.7	2.7
Lysine	1.5	2.5	10.5
Methionine	3.1	3.4	1.0
Threonine	0.1	0.5	1.1
Protease	-	0.2	0.2
Vitamin and mineral premix^1^	2.5	2.5	2.5
Total	1000	1000	1000
**Chemical composition, g/kg**^2^			
Crude protein	210.0	200.0	200.0
Ether extract	54.9	50.4	29.6
Crude fiber	33.9	33.0	27.4
Ash	51.7	50.7	45.5
**Calculated analysis, g/kg**			
Calcium	9.0	9.0	10.0
Phosphorus	7.0	7.0	7.0
Lysine	12.5	12.5	12.5
Methionine+Cystine	10.0	10.0	10.0
Threonine	8.0	8.0	8.0
Tryptophan	2.5	2.3	1.2
Metabolisable energy, Kcal /kg	3100	3100	3100

^1^Supplying per kg feed: 12,000 IU vitamin A, 5,000 IU vitamin D_3_, 80 mg vitamin E, 7 mg vitamin K, 5 mg thiamin, 6 mg riboflavin, 6 mg pyridoxine, 0.02 mg vitamin B_12_, 60 mg niacin, 15 mg pantothenic acid, 1.5 mg folic acid, 0.25 biotin, 10 mg vitamin C, 500 mg choline chloride, 100 mg Zn, 120 mg Mn, 20 mg Fe, 15 mg Cu, 0.2 mg Co, 1 mg I, 0.3 mg Se, and 0.06 mg phytase.

^2^Each diet sample was analyzed in triplicate.

All birds were weighed individually at their placing into the poultry house and later on a weekly basis. All birds were vaccinated against Marek’s disease after hatching and against Newcastle disease, infectious bronchitis and Gumboro during the second week of their life. Feed consumption within each group was recorded during the experimental period and feed conversion ratio was finally calculated. Mortality was also daily recorded.

At the end of the experiments, all birds were slaughtered under commercial conditions and samples of carcasses and gastrointestinal tracts from 4 birds of each replication (i.e. 24 birds per group), were collected for further analysis.

For each subgroup of animals, the samples of breast (*Pectoralis major*) and thigh (*Biceps femoris*) meat without skin were analyzed for moisture, CP and fat content, by near infra-red spectroscopy using a FoodScanTM Lab (FOSS, Denmark). Each sample of the breast or the thigh meat was carefully separated from the skin and the bones, was minced (Cutter K35, Electrolux) and then 200 g of the sample was placed in the instrument.

The alternative diets were also compared regarding their cost per kg of broilers’ weight gain. The feed cost per kg body weight gain was calculated based on the prices of raw materials during the experiments (July, 2015) and according to the Eq (1) which was presented in the work of Choi *et al*.[29].

Feed cost per weight gain (€/kg) = Feed cost (€/kg) x Feed intake per bird (kg) / Weight gain per bird (kg) (1)

### Detection and quantification of bacterial pathogens in small intestine and cecum by polymerase chain reaction

The contents of the cecum and of the ileum of the selected birds were squeezed out into a sterile 50-ml plastic tube. 1 g of the mixed content was transferred in a new sterile 50-ml plastic tube containing 9 ml of sterile PBS and homogenized by vortexing for 3 min. Debris was removed by centrifugation at 500 rpm for 1 min. Furthermore the supernatant was collected and centrifuged at 11000 rpm for 5 min. The pellet was washed twice with PBS and stored at -20°C until DNA extraction. For DNA extraction, the pellet was re-suspended in 50 mM EDTA and treated with lysozyme (Sigma Aldrich Co., St. Louis, MO; final concentration of 10 mg/mL) for 45 min at 37°C. The total bacterial genomic DNA was isolated using a Wizard Genomic DNA purification kit (Promega Corp., Madison, WI) according to the manufacturer’s instructions. The DNA concentration was determined spectrophotometrically. Detection of DNA belonging to *Lactobacilus* spp., *Clostridium perfringens*, *Fusobacterium necroforum*, and *Escherichia coli* was performed by PCR. The targeted genes were amplified by PCR using Taq DNA polymerase in accordance with the supplier's directions (Kapa Biosystems 200 Ballardvale St, Ste 250 Wilmington, MA) The oligonucleotides used for PCRs, the annealing temperatures, and the size of the amplicons are listed in Table 2. The DNA belonging to *Lactobacilus* spp was detected by a 16S rRNA gene. In contrast, the DNA of the rest of the bacterial variables was detected by chromosomal genes. The PCR products were analyzed on 2% agarose gel and quantified using GelQuant.NET software provided by BiochemLab Solutions (biochemlabsolutions.com). Finally the densities of the quantified bands were expressed in arbitrary units [30]. The density of each bank was directly associated to the population of each of the bacterial variables.

**Table 2:** Specifications of the PCR assays applied to the detection of microbial DNA from ileum and cecum samples.

Pathogen	Oligonucleotide primers (5’-3’)	Target gene	Product size	Amplification temperature	References
*Lactobacilus spp*	CTT GTA CAC ACC GCC CGT CA CTC AAA ACT AAA CAA AGT TTC	16S rRNA	250 bp	55°C	[31]
*Clostridium perfringens*	CAACTGCTGGTCCAAATGAAAGCTTTTGAGTCCAAGGGTATG	*cpe*	355 bp	55°C	[32]
*Fusobacterium necrophorum*	ACAATCGGAGTAGTAGGTTCATTTGGTAACTGCCACTGC	*Lkta*	402 bp	59°C	[33]
*Escherichia coli*	CCGATACGCTGCCAATCAGTACGCAGACCGTAGGCCAGAT	*uspA*	884 bp	67°C	[34]

### Intestinal morphology measurements

The morphometric analysis of the small intestine was conducted according to Giannenas *et al*. [35]. During necropsy of the selected birds, the gastrointestinal tract was removed and the small intestine was divided into three parts: 1) duodenum (from the gizzard outlet to the end of the pancreatic loop), 2) jejunum (from the pancreatic loop to Meckel's diverticulum) and 3) ileum (from Meckel's diverticulum to the ileo-caeco-colic junction). Cecum segments were also evaluated. One-cm long segments were taken from the center of each part and fixed in 10% buffered formalin for morphometric studies under light microscopy, with a Nikon microscope coupled with NIS Elements imaging software analysis system (Nikon Eclipse 200, Tokyo, Japan). Images were viewed (4×) to measure morphometric parameters of intestinal architecture. For this purpose, three favorably orientated sections cut perpendicularly from villus enterocytes to the muscularis mucosa were selected from each bird and measurements were carried as follows: villous height (VH) was estimated by measuring the vertical distance from the villous tip to villous-crypt junction level for 10 villi per section; whereas crypt depth (CD) was estimated as the vertical distance from the villous-crypt junction to the lower limit of the crypt for 10 corresponding crypts per section.

### Statistical analysis on broilers’ performance, carcass quality and feed cost

The statistical analysis on broilers’ performance (growth and health i.e. body weight gain, feed intake, FCR, intestinal microbiota populations, gut morphology), carcass quality and feed cost data was performed using the SPSS 21.00 statistical package (IBM SPSS, Inc., Chicago, IL). In every case, the experimental unit of replication was the pen (cage) of birds. The analysis of variance (ANOVA) was performed to examine differences among group means. The homogeneity of the variances was tested by Levene’s test. Values of P≤0.05 were considered significant, whereas values of 0.05<P≤0.10, were considered as trends (tendencies). When significant treatment effects or trends were detected, the Duncan’s test was applied (at significance level 0.05 for significant treatment effects or 0.10 for trends, respectively), in order to determine statistical differences between the means. Mortality was checked by *χ*^*2*^ test.

### Life cycle assessment

#### Goal and scope definition

A partial LCA was conducted, in order to evaluate the environmental performance of three different systems (each one defined by every applied diet). An *attributional ‘cradle-to-farm-gate’* analysis was followed. The term ‘attributional’ implies that the environmental performance is evaluated in a status quo situation (e.g. [36]). The term ‘cradle-to-farm-gate’ refers to the *system boundaries* and indicates that all processes in the production chain up to the sale of broilers’ live-weight (LW) to the slaughterhouse should be considered in the analysis [18, 19].

**Fig 1:**
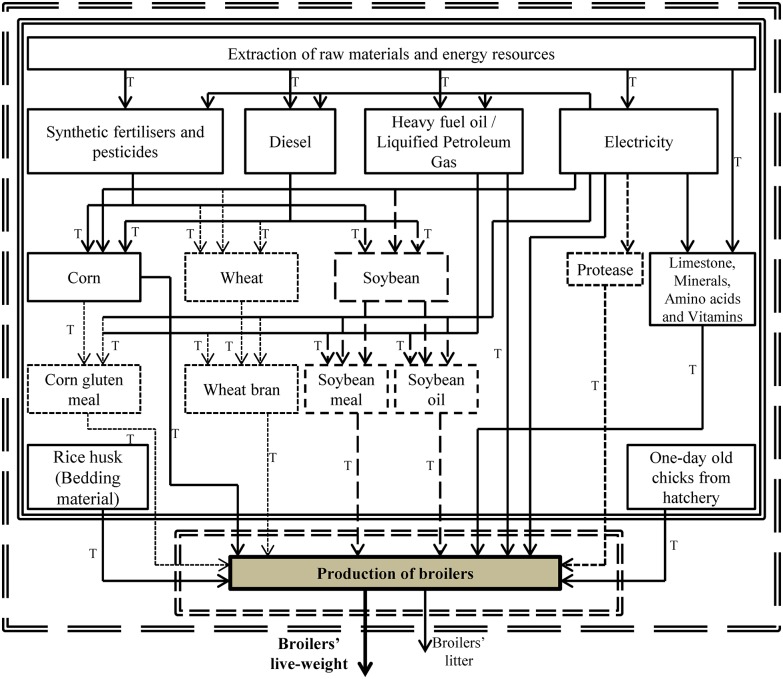
Simplified flowchart indicating the system boundaries for the examined partial life cycle of the production of 1 kg of broilers’ LW. Double bold long-dashed line: system boundaries; double bold short-dashed line: foreground system; double bold continuous line: background system; single bold continuous lines and arrows: processes taking place in all three systems; single bold long-dashed lines and arrows: processes taking place in the ‘Control’ and ‘Soy-Prot’ systems; single bold short-dashed lines and arrows: processes taking place in the ‘Soy-Prot’ and ‘Gluten-Prot’ systems; single regular short-dashed lines and arrows: processes taking place only in the ‘Gluten-Prot’ system; T: Transport processes.

The system boundaries are presented in Fig 1. All transport processes have been taken into account in the analysis. However, the partial life cycles of buildings and machinery as well as those of cleaning and disinfection chemicals and veterinary medicines, have not been taken into consideration due to lack of data and due to their expected low contribution to the total environmental impacts of the system [19, 22]. Moreover, as the purchase of the diets’ additives was less than 1% of the total mass, they were excluded from the analysis, assuming that they will have a very low contribution [18]. The *functional unit (FU)* is defined as ‘1 kg of broiler LW at the farm gate’. The *time boundary* for collecting primary data for the *foreground system* (i.e. the broilers’ production process in the poultry house and the transport processes of material inputs to the farm) (Fig 1) was equal to the duration of the experiment (i.e. 42 days).

Ten *environmental impact categories* were assessed in the context of this LCA, namely depletion of abiotic resources, photochemical oxidation, acidification, eutrophication, cumulative energy demand, land use (occupation and transformation), global warming, human toxicity (cancer and non-cancer effects), freshwater ecotoxicity and water scarcity.

*Economic allocation* was applied to partition the environmental burden between the co-products in all the relevant processes of the *background system* (Fig 1) [20, 21]. Economic allocation is considered as a suitable approach to distribute the environmental burden to process’ co-products when their origin is the feed industry [37]. For the Control and the Soy-Prot systems, these processes included the soybean crushing, the rice husk production and the breeding stage which results in the birth of one-day chicks. For the Gluten-Prot system these processes included the CGM, the wheat bran, the rice husk and the breeding production. For transport processes, it was assumed that the different means of transport were solely loaded with one material and that an additional 20% of the emissions of the first trip corresponds to the return trip [38]. In the *foreground system*, there were no multi-functionality issues since the litter produced was not useful within the system boundaries [18]. It was not processed for energy producing or fertilizing purposes within the farm but it was collected and stored in open litter piles for the whole year. Therefore, the environmental burden can be entirely attributed to the production of the broilers’ live-weight in all the systems for comparison.

#### Life cycle inventory compilation

Regarding the compilation of the life cycle inventory (LCI), apart from diets’ composition, other broiler related parameters were taken into account, such as the quantity of the diet consumed, the broilers’ mortality percentage, the weight of the new-born chicks when purchased, the broilers’ final weight before sale, the daily weight-gain of broilers and the quantity of bedding material purchased (i.e. 2 kg/bird/production cycle). The losses of feed constituents during the on-farm rations’ formation and during the feed consumption were assumed equal to 0.5% w/w and 0.1% w/w, respectively. The volume of the water consumed by the broilers during the experiments was considered equal to twice the quantity of the feed consumed in liters. The electricity and the heat consumption were estimated 0.1021 and 0.3086 kWh per kg of produced broiler, respectively.

*Emissions* of gases from the broiler house but also from the litter piles were also taken into account in the analysis. Enteric methane (CH_4_) emissions from broilers were estimated by selecting an emission factor equal to 0.015 g CH_4_/broiler/year [18]. For CH_4_ emissions due to manure deposition in the house, a Tier 2 IPCC approach was implemented [39]. Moreover, daily manure production was considered equal to 0.08 kg/day/kg of broiler [40], while the ash content of the excreted manure was taken equal to 10% [18]. Ammonia (NH_3_), nitrous oxide (N_2_O) (direct and indirect), nitric oxide (NO) and nitrogen (N_2_) emissions were estimated by applying a Tier 2 EMEP/EEA approach [41] and a Tier 1 IPCC approach [39]. The default values / methodologies proposed for all emission factors in the respective guidelines were utilized, apart from the nitrogen (N) retention from broilers, the value of which (0.602 kg N/kg N intake) was taken from the related ASAE standard [42]. In this analysis, N related indicators, processes and flows may be slightly overestimated as, following the methodology also adopted by [43], the possible effect of the protease on nitrogen retention of broilers was not considered due to data unavailability. For CO_2_ emissions due to LPG combustion, a country-specific emission factor (Tier 2 approach) was used [44].

For the background system, secondary data was acquired from LCI databases available in the software package SimaPro, v. 8.0.4.26 PhD [45]. The production of all the plant-based feed ingredients (i.e. corn, SBM, soybean oil, CGM and wheat bran) as well as the production of rice husk and all transport processes, were modeled by using the Agri-footprint v.1database [38, 46]. GHG emissions due to direct land use change (LUC) were considered for soybean production (Direct Land Use Change Assessment Tool v. 2014–1 [46]. The chicken breeding processes that result in the production of the one-day old chicks, were also modeled up to the great grandparent generation [18] with the aforementioned database. The modeling of the LPG, electricity and limestone production processes was realized by using the Ecoinvent v.3 database [47]. In addition, the diesel and heavy fuel oil consumption mixes provided in the ELCD v.3 database [48] were utilized.

All plant-based feed ingredients including the bedding material are domestically produced, except for the 90% of SBM [49]. For all domestic production processes, it was attempted to adjust the existing models to the Greek conditions to the furthest possible extent (e.g. representative avg. yields for corn and wheat production [50], use of steam from heavy fuel oil as the major carrier for heat in the feed industry). The synthetic fertilizers used for corn, wheat and rice production were assumed to be entirely imported from North-Western Europe, whereas the production of the pesticides used was not taken into account due to lack of data. SBM was assumed to be imported in equal quantities from Brazil and Argentina. Additionally, for all the domestic production processes of the examined life cycles, the medium voltage electricity consumption mix in Greece for 2013 was used [51]. Concerning transport processes, it was assumed that all materials were directly transported from their production site to the broiler farm (except LPG, which was transported to the farm via a gas station). The transport distances, together with the means of transport and the origin of the input materials are presented in Table 3.

**Table 3:** Transport distances to the broiler farm.

Material	Systems	Origin	Transport distance (km)^1^	Means of transport
One-day chicks	All	Greece	10	Truck (<10t)
Corn	All	Greece	150	Truck (<10t)
Limestone	All	Greece	150	Truck (<10t)
Rice husk	All	Greece	100	Truck (<10t)
LPG	All	Greece	110	Truck (>20t), Truck (<10t)
Soybean meal	Control, Soy-Prot	Greece	550	Truck (<10t)
		Argentina	13084	Sea ship (80000 DWT), Truck (<10t)
		Brazil	11362	Sea ship (80000 DWT), Truck (<10t)
Crude soybean oil	Control, Soy-Prot	Greece	550	Truck (<10t)
Corn gluten meal	Gluten-Prot	Greece	250	Truck (<10t)
Wheat bran	Gluten-Prot	Greece	250	Truck (<10t)

^1^All domestic distances are estimated by having taken into account the average distances between possible realistic production sites and the broiler farm. For imported soybean meal, the distances are estimated according to [1].

The economic allocation factors for the milling industry (i.e. rice husk, CGM and wheat bran) included in the Agri-footprint database were exploited [52, 53]. However, for the Crushing Industry, the economic allocation factors were based on the 5-year (2010–2014) average world price of SBM (320.3 euro/ton) provided by the World Bank [54], by assuming that crude soybean oil costs double the price of SBM while soybean hulls half its price [55]. Finally, in the breeding stage, the economic allocation between hens for slaughter, eggs for consumption and eggs for hatchery was applied by considering the 5 year average (2010–2014) producer prices per kg of live weight of chicken (1.52 euro/kg) in Greece and per kg of egg (1.29 euro/kg) [56].

#### Life cycle impact assessment

Mid-point life cycle impact assessment (LCIA) [57] was applied in this study. The LCIA methods used for the conversion of the LCI results to impact categories, together with the relevant Environmental Impact Category Indicators (EICIs), are listed in Table 4. All methods are embedded in the software package SimaPro, v. 8.0.4.26 PhD [45, 58].

**Table 4:** Life cycle impact assessment (LCIA) methods for the evaluation of the environmental impact category indicators (EICI) in this study.

EICI	LCIA method
ADP, AP, EP	CML-IA baseline v. 3.02
POP, ALO, NLT	ReCiPe Midpoint (H) v. 1.11
CED	Cumulative Energy Demand v. 1.09
GWP_100_	IPCC 2013 GWP 100a v. 1
HTPc, HTPnc, FWETP	USEtox (recommended + interim) v. 1.04
WDI	Berger *et al*. 2014 (Water Scarcity) v. 1.00

## Results

### Effect of diets on broilers’ growth performance, health, carcass traits and economics

The results of this trial on growth performance are illustrated in Table 5. As it is shown, after the second week and throughout the rest of the trial, the body weight values in Gluten-Prot group were significantly lower or tended to be lower than Control and Soy-Prot groups. In addition, feed intake and FCR values did not differ significantly (P>0.05) among the experimental groups. Overall feed intake for Gluten-Prot group showed a decreasing trend (P = 0.055) compared to control group, whereas FCR values tended to be worse for the Gluten-Prot group only during the first growth period. Mortality values were 1.1% for each group and did not differ significantly (P>0.05) between the groups.

**Table 5:** Influence of diets on body weight, feed intake and feed conversion ratio of broiler chickens.

Body weight, kg	Group			SEM^2^	P
	Control^1^	Soy-Prot^1^	Gluten-Prot^1^		
Day 0	0.044	0.044	0.044	0.0001	0.628
Day 7	0.130	0.128	0.130	0.0020	0.868
Day 14	0.307 ^b^	0.311 ^b^	0.268 ^a^	0.0054	0.001
Day 21	0.799 ^y^	0.790 ^y^	0.740 ^x^	0.0120	0.091
Day 28	1.352 ^b^	1.299 ^b^	1.182 ^a^	0.0235	0.003
Day 35	2.021 ^b^	2.005 ^b^	1.815 ^a^	0.0293	0.001
Day 42	2.424 ^y^	2.389 ^y^	2.259 ^x^	0.0325	0.084
**Feed intake, kg**					
Days 0–14	0.507	0.483	0.462	0.0092	0.125
Days 15–28	1.050	1.052	1.025	0.0128	0.651
Days 29–42	2.199	2.165	2.152	0.0152	0.447
Days 0–42	3.757 ^y^	3.700 ^xy^	3.639 ^x^	0.0207	0.055
**FCR**^3^					
Days 0–14	1.937 ^xy^	1.804 ^x^	2.065 ^y^	0.0454	0.053
Days 15–28	1.009	1.070	1.130	0.0268	0.184
Days 29–42	2.056	1.995	2.048	0.0516	0.884
Days 0–42	1.579	1.581	1.653	0.0243	0.389

n = 6 replications per group.

^1^ Control: Soybean meal based diet; Soy-Prot: Soybean meal based diet with protease addition; Gluten-Prot: Gluten meal based diet with protease addition.

^2^ SEM: standard error of the mean.

^3^ FCR: Feed conversion ratio (kg of feed / kg of body weight gain)

^a,b^ Mean values in a row with no superscript in common differ significantly (P≤0.05).

^x,y^ Mean values in a row with no superscript in common have a tendency to differ (P≤0.10).

In our work, meat quality characteristics of both breast and thigh in all three experimental groups were within acceptable values (Table 6). The CP content in the breast was found to be significantly (P = 0.005) lower in the Soy-Prot group than in the Gluten-Prot group; however no significant differences were found either in moisture and fat content of breast tissue or in moisture, protein and fat of thigh tissue.

**Table 6:** Influence of diets on broiler chickens breast and thigh meat chemical composition.

	Group			SEM^2^	P
	Control^1^	Soy-Prot^1^	Gluten-Prot^1^		
**Breast, %**					
Moisture	71.0 ^x^	71.6 ^xy^	71.8 ^y^	0.1	0.074
Crude fat	6.8	6.9	6.6	0.1	0.403
Crude protein	21.6 ^ab^	21.0 ^a^	21.9 ^b^	0.1	0.005
**Thigh, %**					
Moisture	71.9	70.9	71.6	0.3	0.283
Crude fat	9.5	9.3	9.0	0.2	0.410
Crude protein	19.5	19.3	19.8	0.1	0.387

n = 6 replications per group

^1^ Controls: Soybean meal based diet; Soy-Prot: Soybean meal based diet with protease addition; Gluten-Prot: Gluten meal based diet with protease addition.

^2^ SEM: standard error of the mean.

^a,b^ Mean values in a row with no superscript in common differ significantly (P≤0.05).

^x,y^ Mean values in a row with no superscript in common have a tendency to differ (P≤0.10).

Table 7 shows the feed cost and the economic benefit, calculated as feed cost per kg weight gain. The cost of feed intake per kg of body weight gain was significantly (P = 0.018) higher for the Gluten-Prot group, compared to the Soy-Prot group during the period from 0 to14 d and tended (P = 0.087) to be higher during the period from 15 to 28 d, for the Gruten-Prot group, compared to the control Group. No significant differences in cost were found during the periods from 29 to 42 d and overall (0 to 42 d).

**Table 7:** Influence of diets on feeding cost of broiler chickens.

	Group			SEM^2^	P
	Control^1^	Soy-Prot^1^	Gluten-Prot^1^		
**Feed cost, €/kg**	0.3049	0.3034	0.3124		
**Feed cost per weight gain**					
Days 0–14	0.5905 ^ab^	0.5473 ^a^	0.6450 ^b^	0.0150	0.018
Days 15–28	0.3075 ^x^	0.3247 ^xy^	0.3531 ^y^	0.0086	0.087
Days 29–42	0.6268	0.6052	0.6397	0.0163	0.707
Days 0–42	0.4814	0.4796	0.5164	0.0082	0.114

n = 6 replications per group

^1^ Control: Soybean meal based diet; Soy-Prot: Soybean meal based diet with protease addition; Gluten-Prot: Gluten meal based diet with protease addition.

^2^ SEM: standard error of the mean.

^a,b^ Mean values in a row with no superscript in common differ significantly (P≤0.05).

^x,y^ Mean values in a row with no superscript in common have a tendency to differ (P≤0.10).

### Intestinal microbiota populations

The results for microbial populations in the ileum and the cecum are shown in Table 8. Densities of the quantified bands expressed in arbitrary units (AU) reflect to the amount of genetic material of each of the 4 pathogens that was detected by PCR in our study. In the ileum, DNA from *C*. *perfringens* was detected in statistically lower AU in the Gluten-Prot group compared to the other groups. Furthermore, both DNA from *C*. *perfringens* and *F*. *necrophorum* were also statistically lower in cecum in both groups supplemented with protease and fed lower protein levels compared to control group. No significant differences were noted for *Lactobacillous* spp. and *E*. *Coli* in either ileum or cecum among the experimental groups.

**Table 8:** Results per microbial pathogen and experimental group recorder by PCR and expressed in arbitrary units (AU).

	Group			SEM^2^	P
	Control^1^	Soy-Prot^1^	Gluten-Prot^1^		
**Ileum**					
*Lactobacillus* spp.	12.455	12.874	12.293	0.192	0.464
*Clostridium perfringens*	1.151 ^b^	1.089 ^b^	1.017 ^a^	0.017	0.002
*Fusobacterium necroforum*	1.954	1.888	1.953	0.018	0.245
*Escherichia coli*	1.420	1.387	1.355	0.017	0.298
**Cecum**					
*Lactobacillus* spp.	1.181	1.186	1.182	0.001	0.274
*Clostridium perfringens*	5.162 ^b^	4.723 ^a^	4.686 ^a^	0.089	0.042
*Fusobacterium necroforum*	2.717 ^b^	2.379 ^a^	2.271 ^a^	0.052	0.001
*Escherichia coli*	1.182	1.158	1.155	0.006	0.158

n = 6 replications per group

^1^ Controls: Soybean meal based diet; Soy-Prot: Soybean meal based diet with protease addition; Gluten-Prot: Gluten meal based diet with protease addition.

^2^ SEM: standard error of the mean.

^a,b^ Mean values in a row with no superscript in common differ significantly at P≤0.05.

### Intestinal morphology

Intestinal architecture was not significantly influenced by the different diets in terms of villus height, crypt depth and villus height to crypt depth ratio in the duodenum, jejunum and ileum (Table 9). No morphologic lesions or any inflammatory responses were found in the examined intestinal parts of both cecum and small intestine.

**Table 9:** Influence of diets on intestinal morphology (villus height and crypt depth) of broiler chickens.

Villus Height, mm	Group			SEM^2^	P
	Control^1^	Soy-Prot^1^	Gluten-Prot^1^		
Duodenum	1703.48	1668.93	1666.29	25.28	0.808
Jejunum	1292.44	1320.29	1353.30	19.91	0.471
Ileum	740.49	703.43	732.17	12.02	0.430
**Crypt Depth, mm**					
Duodenum	183.40	180.65	183.32	6.08	0.979
Jejunum	138.06	128.43	133.60	4.08	0.641
Ileum	115.72	108.33	116.89	4.35	0.696
**Villus height to crypt depth ratio**					
Duodenum	9.85	9.65	9.45	0.36	0.799
Jejunum	9.56	10.68	10.33	0.31	0.332
Ileum	6.76	6.80	6.40	0.23	0.759

n = 6 replications per group

^1^ Controls: Soybean meal based diet; Soy-Prot: Soybean meal based diet with protease addition; Gluten-Prot: Gluten meal based diet with protease addition.

^2^ SEM: standard error of the mean.

No significant (P>0.05) differences were found.

### Life cycle assessment

#### Environmental performance

The potential environmental impacts per year corresponding to 1 kg of broilers’ LW produced in the three different partial life cycles are presented in Table 10. The values for ADP, AP, EP, POP, ALO, NLT, CED, GWP_100_, HTPc, HTPnc, FWETP and WDI ranged between 1.00 and 1.51 (×10^−6^) kg Sb eq., 0.0287 and 0.0324 kg SO_2_eq, 0.0175 and 0.0178 kg PO_4_^3-^eq, 3.90 and 5.40 (×10^−3^) kg NMVOC eq, 1.86 and 3.55 m^2^∙year, 0.05 and 5.16 (×10^−2^) m^2^, 14.67 and 15.39 MJ, 1.63 and 4.21 kg CO_2_eq, 4.45 and 6.47 (×10^−8^) CTUh, 2.59 and 4.26 (×10^−6^) CTUh, 16.31 and 17.52 CTUe and 0.24 and 0.367 m^3^, respectively, per kg broiler LW at the farm gate and year. In 11 out of 12 category indicators, the Soy-Prot and Gluten-Prot systems showed the same trend, as both performed better than the Control system in 9 (ADP, AP, EP, POP, ALO, NLT, GWP_100_, HTPnc, FWETP) and worse than the Control system in 2 category indicators (HTPc, WDI). In one category (CED), the Soy-Prot system performed better than the Control system, while the Gluten-Prot system worse. The differences between the Gluten-Prot system and the Control system were larger than the differences between the Soy-Prot system and the Control system in all impact categories except for eutrophication (EP).

**Table 10:** Potential environmental impacts per kg of broiler LW at the farm gate and year for the three different systems examined.

EICI	Units (per kg broiler LW and year)	System^a^		
		Control	Soy-Prot	Gluten-Prot
ADP	(×10^−6^) kg Sb eq.	1.51	1.45	1.00
AP	(×10^−2^) kg SO_2_ eq.	3.24	3.12	2.87
EP	(×10^−2^) kg PO_4_^3-^ eq.	1.78	1.75	1.75
POP	(×10^−3^) kg NMVOC eq	5.40	5.20	3.90
ALO	m^2^∙year	3.55	3.35	1.86
NLT	(×10^−2^) m^2^	5.16	4.64	0.05
CED	MJ	14.92	14.67	15.39
GWP_100_	kg CO_2_ eq.	4.21	3.92	1.63
HTPc	(×10^−8^) CTUh	4.45	4.54	6.47
HTPnc	(×10^−6^) CTUh	4.26	4.06	2.59
FWETP	CTUe	17.52	17.20	16.31
WDI	(×10^−1^) m^3^	2.40	2.51	3.67

^a^ All values were rounded to the 2^nd^ decimal digit

#### Contribution of substances and processes

The potential contribution of substances and processes to the EICIs’ values for each one of the three systems was also examined. Tables 11 and 12 show the most important contributing (≥ 10% to the total indicator value) flows and processes, respectively. Table 11 shows that for half of the EICIs only one major contributing flow was identified, meaning that every EICI was almost entirely determined by the corresponding major contributing flow for all the systems. Two major contributing flows were detected for AP, three for EP, CED and GWP_100_ and four for FWETP. For two of these EICIs (i.e. AP and EP), the flows showed similar behavior of significance for the three systems. Furthermore, for two EICIs (i.e. CED and GWP_100_) the flows showed similar behavior of significance for the systems Control and Soy-Prot but different behavior for the Gluten-Prot system. There was only one EICI (i.e. FWETP) for which the flows of all three systems exhibited a different behavior of significance. For example, as far as GWP_100_ is concerned, for both the Control and Soy-Prot systems, the emissions of CO_2_ from direct land transformation were the most important flow (62.3% and 60.2%, respectively), followed by the emissions of CO_2_ from fossil fuels (23.8% and 25.1%, respectively) and the emissions of N_2_O (11.5% and 12.1%, respectively). However, for the Gluten-Prot system, the emissions of CO_2_ from fossil fuels constituted the dominant flow (62.8%), followed by the emissions of N_2_O (29.4%), while the emissions of CO_2_ due to direct land transformation contributed extremely low (1.7%) to the GWP_100_ value.

**Table 11:** Major contributing flows (≥10% contribution) and their potential contribution to the total environmental impact category indicator (EICI) values for the three systems studied.

EICI	Units (per kg broiler LW)	Contributing flow	System / Value^a^ (% Contribution^b^)		
			Control	Soy-Prot	Gluten-Prot
ADP	(×10^−6^) kg Sb	Phosphorus (P) (raw material)	1.45 (95.8)	1.39 (95.6)	0.93 (92.5)
AP	(×10^−2^) kg SO_2_ eq.	Ammonia (NH_3_) (air)	2.70 (83.5)	2.60 (83.4)	2.26 (78.7)
		Sulfur dioxide (SO_2_) (air)	0.29 (8.8)	0.28 (9.0)	0.44 (15.2)
EP	(×10^−3^) kg PO_4_^3-^ eq.	Nitrate (NO_3_^-^) (water)	7.12 (39.9)	7.04 (40.3)	6.88 (39.3)
		Ammonia (NH_3_) (air)	5.91 (33.2)	5.69 (32.5)	4.94 (28.2)
		Phosphate (PO_4_^3-^) (water)	2.02 (11.3)	2.06 (11.8)	2.98 (17.0)
POP	(×10^−3^) kg NMVOC eq	Nitrogen oxides (NO_x_) (air)	4.16 (76.7)	3.99 (76.8)	2.79 (72.3)
ALO	m^2^∙year	Arable land (raw material)	3.54 (99.9)	3.35 (99.9)	1.86 (99.8)
NLT	(×10^−2^) m^2^	Transformation from forest (raw material)	5.16 (100.0)	4.64 (100.0)	0.05 (99.9)
CED	MJ	Crude oil (raw material)	7.93 (53.2)	7.71 (52.6)	7.47 (48.6)
		Natural Gas (raw material)	3.15 (21.1)	3.09 (21.1)	2.85 (18.5)
		Lignite (raw material)	2.81 (18.9)	2.88 (19.6)	4.17 (27.1)
GWP_100_	kg CO_2_ eq.	Carbon dioxide (CO_2_), Land transformation (air)	2.62 (62.3)	2.36 (60.2)	0.03(1.7)
		Carbon dioxide (CO_2_), Fossil (air)	1.00 (23.8)	0.98 (25.1)	1.02(62.8)
		Nitrous oxide (N_2_O) (air)	0.48 (11.5)	0.47 (12.1)	0.48 (29.4)
HTPc	(×10^−8^) CTUh	Chromium VI (Cr(VI)) (water)	3.90 (87.7)	3.99 (87.9)	5.78 (89.3)
HTPnc	(×10^−6^) CTUh	Zinc (Zn) (Soil)	3.80 (89.2)	3.60 (88.8)	2.11 (81.4)
FWETP	CTUe	Chlorpyrifos^c^ (Soil)	3.38 (19.3)	3.03 (17.6)	0.03(0.2)
		Zinc (Zn) (Water)	3.05 (17.4)	3.09 (18.0)	4.17 (25.6)
		Zinc (Zn) (Soil)	1.92 (11.0)	1.82 (10.6)	1.07(6.6)
		Alachlor^f^ (Soil)	1.49 (8.5)	1.57 (9.1)	2.21 (13.6)
WDI	m^3^	n/a^d^	n/a^d^	n/a^d^	n/a^d^

^a^ All values were rounded to the 2^nd^ decimal digit

^b^ All values were rounded to the 1^st^ decimal digit

^c^ The total quantity of pesticides applied is assumed to be emitted directly to the soil [38]

^d^ Not applicable to water depletion index, as water is the only relevant flow, either used as raw material or emitted to the ecosphere

**Table 12:** Major contributing processes (≥10% contribution) and their potential contribution to the total environmental impact category indicator (EICI) values for the three systems studied.

EICI	Units (per kg broiler LW)	Contributing process (Origin)	System / Value^a^ (% Contribution^b^)		
			Control	Soy-Prot	Gluten-Prot
ADP	(×10^−6^) kg Sb	Phosphate rock fertilizer production (WE^c^)	1.45 (95.8)	1.39 (95.6)	0.93 (92.5)
AP	(×10^−2^) kg SO_2_ eq.	Foreground system (Gr^c^)	1.14 (35.2)	1.08 (34.8)	1.13 (39.2)
		Soybean production (Ar^c^, Br^c^, US^c^)	0.74 (22.9)	0.67 (21.3)	0.01(0.2)
		Corn production (Gr^c^)	0.36 (11.1)	0.38 (12.2)	0.53 (18.6)
EP	(×10^−3^) kg PO_4_^3-^ eq.	Soybean production (Ar^c^, Br^c^, US^c^)	4.28 (24.0)	3.84 (22.0)	0.04(0.2)
		Foreground system (Gr^c^)	3.90 (21.9)	3.71 (21.2)	3.86 (22.0)
		Corn production (Gr^c^)	3.88 (21.8)	4.09 (23.4)	5.76 (32.9)
		Lignite mining (Gr^c^)	2.05 (11.5)	2.10 (12.0)	3.04 (17.4)
POP	(×10^−3^) kg NMVOC eq	Diesel combustion in machinery (All^c^)	1.61 (29.7)	1.58 (30.5)	1.40 (36.3)
		Transport, truck (<10t) (Gr^c^)	1.12 (20.7)	1.07 (20.6)	0.75 (19.3)
		Transport, sea ship (80000 DWT) (All^c^)	0.68 (12.5)	0.61 (11.8)	0.02(0.4)
ALO	m^2^ ∙year	Soybean production (Ar^c^, Br^c^, US^c^)	2.34 (66.0)	2.10 (62.5)	0.02(1.2)
		Corn production (Gr^c^)	0.85 (23.9)	0.89 (26.7)	1.26 (67.5)
		Wheat production (Gr^c^)	0.14 (4.1)	0.15 (4.4)	0.32 (17.1)
NLT	(×10^−2^) m^2^	Soybean production (Ar^c^, Br^c^)	5.15 (99.9)	4.64 (99.9)	0.05 (93.4)
CED	MJ	Diesel production and transport (All^c^)	5.12 (34.3)	4.94 (33.7)	3.68 (23.9)
		Lignite mining (Gr^c^)	2.80 (18.8)	2.87 (19.6)	4.16 (27.0)
		Steam from heavy fuel oil (Gr^c^)	0.29 (1.9)	0.28 (1.9)	1.61 (10.5)
GWP_100_	kg CO_2_ eq.	Soybean production (Ar^c^, Br^c^)	2.76 (65.6)	2.49 (63.5)	0.03(1.6)
		Foreground system (Gr^c^)	0.24 (5.7)	0.23 (5.9)	0.24 (14.7)
		High voltage electricity from lignite (Gr^c^)	0.17 (4.0)	0.17 (4.4)	0.25(15.5)
		Corn production (Gr^c^)	0.14 (3.3)	0.15 (3.8)	0.21 (12.8)
HTPc	(×10^−8^) CTUh	Lignite mining (Gr^c^)	4.03 (90.6)	4.12 (90.8)	5.97 (92.3)
HTPnc	(×10^−6^) CTUh	Soybean production (Ar^c^, Br^c^, US^c^)	2.59 (60.8)	2.32 (57.2)	0.02(0.9)
		Corn production (Gr^c^)	1.02 (24.0)	1.08 (26.6)	1.52 (58.6)
		Wheat production (Gr^c^)	0.20 (4.8)	0.21 (5.1)	0.45 (17.5)
FWETP	CTUe	Soybean production (Ar^c^, Br^c^, US^c^)	6.60 (37.7)	5.93 (34.5)	0.06(0.4)
		Corn production (Gr^c^)	4.12 (23.5)	4.34 (25.2)	6.11 (37.5)
		Lignite mining (Gr^c^)	3.68 (21.0)	3.76 (21.9)	5.45 (33.4)
		Lignite ash treatment (Gr^c^)	1.16 (6.6)	1.19 (6.9)	1.72 (10.5)
WDI	(×10^−1^) m^3^	Corn production (Gr^c^)	2.00 (81.7)	2.10 (82.4)	2.90 (79.4)
		Wheat production (Gr^c^)	0.30 (11.5)	0.30 (11.2)	0.60 (16.6)

^a^All values were rounded to the 2^nd^ decimal digit

^b^All values were rounded to the 1^st^ decimal digit

^c^ The origin is specified. All: all production locations and imports by ship to Greece, Gr: Greece, Ar: Argentina, Br: Brazil, US: United States of America, WE: Western Europe

Table 12 shows that for few of the EICIs (i.e. ADP, NLT, HTPc) only one major contributing process was identified. For the majority of the EICIs, their values were determined by two or more processes for all the systems. Two major contributing processes were detected for WDI, three for AP, POP, ALO, CED and HTPnc and four for EP, GWP_100_ and FWETP. For all the EICIs with more than one major contributing process except for EP, the processes showed similar behavior of significance for the Control and Soy-Prot systems. The Gluten-Prot system’s processes exhibited similar behavior of significance with the other two systems only for POP and WDI. For example, as far as the GWP_100_ is concerned, the soybean production was the most significant process contributing 65.6% and 63.5% for the Control and the Soy-Prot system, respectively, while the contributions of the foreground system, the high voltage electricity production from lignite and the corn production were lower than 6% for both systems. On the contrary, in the Gluten-Prot system, the contribution of the soybean production was extremely low (i.e. 1.6%), while the contributions of the foreground system, the high voltage electricity production from lignite and the corn production to the GWP_100_ value, were 14.7%, 15.5% and 12.8%, respectively.

## Discussion

### Effect of diets on broilers’ growth performance, health, carcass traits and economics

The results regarding the effects of protease on chicken growth performance are in agreement with the results produced by other experiments [12, 13] where SBM was the main proteinaceous feedstuff in both experimental diets. According to these findings, a reduction of protein feed level by 10% could be compensated by protease inclusion, coupled with other feed additives like compounds of plant essential oils and organic acids [12, 13]. In the current trial, dietary groups with recommended protein levels, together with protease would not be of interest for two reasons. It is questionable, whether the extra cost of protease would be compensated by higher production, and for this reason it is usually not recommended by protease producers [13, 43, 59, 60]. In addition, no possible benefits would be expected for LCA, as proteinaceous feedstuffs should be used at higher levels.

In the Gluten-Prot group a lower body weight was attained, compared to the other two groups. One explanation might be the notable difference in tryptophan content in the feeds, and possibly for other essential amino acids. Despite the effort made to equalize lysine, methionine and threonine, in the Gluten-Prot group, reduction in tryptophan and possible reduction in other essential or non-essential amino acids, as well as reduction in overall protein content by 10%, could not be fully equalized by the addition of protease. However, no differences were detected in feed intake, feed conversion and mortality values. In broiler chickens, supplements of methionine, lysine and tryptophan may be more efficiently utilized when provided in pure form rather than as components of intact protein, particularly at the higher dietary crude protein concentrations. The reduced utilization of protein-bound amino acids observed in broilers has not been fully explained, whereas, lysine and threonine supplements are effective in raising the amino acid profile of barley-based diets to the ideal balance, but frequency of feeding may determine efficiency of utilization [1, 61].

The reduction of SBM in feed inclusion at lower level along with protease or substitution of SBM by CGM and protease did not change the quality of the meat. Moreover, based on the available literature, the economic benefit of substituting SBM by CGM with or without protease has not been estimated in broilers. Comparing the feed cost of Control group vs Soy-Prot group, it can be concluded that the inclusion of protease actually decreased the feed cost per kg, compared to a standard feed, because the additional cost of the enzyme was compensated by the reduction of total crude protein in the feed. The substitution of SBM by CGM marginally influenced the feed cost per kg. Nevertheless, the overall feed cost per weight gain was comparable between the groups. Chicken meat is a cheap and nutritional protein source, consumed all over the world without any special restrictions (i.e. religious or others). Rising production cost due to changes in feed prices could make chicken meat production unaffordable. Also, profound changes in the meat appearance or organoleptic characteristics or chemical composition could make this meat undesirable for the consumer. For example, increase in fat and decrease in lean meat is considered “unhealthy” according to modern diet recommendations. Accordingly, any change in the broiler production systems should not negatively affect either the cost or the meat quality.

### Intestinal morphometry

In the present study, no significant changes were noted on intestinal morphometry. Also no lesions were found to suggest underlying clinical or subclinical inflammatory responses in birds’ gastrointestinal tract. All birds were healthy, without any deaths or signs of clinical diarrhea. The structure of the intestinal mucosa can reveal some information on gut health, and the intestinal villus is regarded as a marker for the capacity of the bird to absorb nutrients from the feed. Longer villi are typically associated with excellent gut health and high absorptive efficiency, whereas shorter villi and deeper crypts correlate with increased counts of pathogenic bacteria in the gastrointestinal tract [62, 63].

### Intestinal microbiota population

Dietary supplementation of protease and reduced levels of SBM or CGM shifted microbiota populations by decreasing clostridia and retaining lactobacillus loads compared to control diet. Regarding the possible effects of CGM in gut function and microbial balance, it has been reported that some of the included substances, for example non-starch polysaccharides fiber or other constituents may modify the gastrointestinal tract fermentation process, directly affecting the digesta composition and the microflora balance in monogastric animals, i.e., swine [64], poultry [65, 66] and fish [67]. In the present study, reduced levels of feed protein in combination with protease did not alter *Lactobacillus spp*. populations but reduced *C*. *perfringens* population, which is the etiologic agent of necrotic enteritis and clostridial diarrhea. Due to the danger that *C*. *perfringens* poses, several strategies have been implicated to reduce clostridial counts in the chicken gut [68].

### Life cycle assessment

#### Uncertainty in the environmental impacts

The analysis performed is dependent on LCI databases which were not compiled for Greek conditions concerning the background system, and uses the default methodologies for national inventory compilation with non-site (or country) specific emission factors for the estimation of emission outputs of the foreground system. Inevitably, uncertainty is caused regarding the LCI results, which is propagated to the results of the EICIs. The various sources and types of uncertainty in LCA as well as the ways of incorporating it in the environmental impact results, have been discussed in the literature (e.g. [69], [70]). The result of an uncertainty assessment regarding an EICI could be expressed as a frequency distribution which approximates its probability distribution, after having performed a Monte Carlo probabilistic simulation [69]. Thus, standard statistics could be used and a quest for significant differences between the EICI results of the different systems tested could be attempted. In case of large uncertainties in the EICIs (possible especially if the uncertainty in all LCI data is considered), drawing solid conclusions with respect to the environmental superiority of a system may be difficult or even impossible [71] and proper sensitivity analysis may be required to find the parameters with the largest influence in the uncertainty of an EICI [72].

Performing such an uncertainty assessment was however out of the scope of this paper. In this study, and in accordance to the objectives initially set, it was chosen to use LCA as a descriptive method and the results which are presented are the deterministic values which were received as outputs of the software package SimaPro, v. 8.0.4.26 PhD [45, 58]. These results are considered as initial, indicative figures regarding the environmental performance of the studied systems. Nevertheless, the current analysis provides in depth investigation of the three systems of interest as well as identification of their environmental hot spots.

#### Comparison to literature results

Few studies have been conducted to comparatively assess the environmental impacts of a broiler production system when broilers are fed with different diets. The study of Leinonen *et al*.[24] showed that the application of a diet in broilers, in which soybeans are partly replaced with beans, peas and sunflower meal (all of European origin), reduced (up to 8%) the GWP_100_ (in all cases except for the sunflower-based diet), the AP (up to 14.5%) and the EP (up to 2.5%) compared to a soybean-based diet. Maximum reductions were found for all indicators when broilers’ diet was enriched with peas, the soybeans content was reduced by 32% and the soybean oil content was increased by 7%. Furthermore, Bundgaard *et al*.[25] compared the GWP_100_ of broiler production for two scenarios of feed formulations, one including a mixture of digestibility-improving enzymes (XAP: xylanase, amylase, protease), and one without it. The diet enriched with XAP was composed with 3.7% less SBM compared to the diet without XAP. The authors estimated that the application of the diet enriched with XAP triggered a reduction of 5–9% in GWP_100_. In addition, Leinonen and Williams[26] estimated the GWP_100_, the AP and the EP in two broiler production systems defined by a soybean-based diet and by an equivalent diet supplemented with the protease Ronozyme^®^ ProAct, whose basic ingredients were wheat (increased by 5%), corn (kept constant), soybean 48 (reduced by 9.6%), rapeseed meal (kept constant) and soybean oil (reduced by 9%). The maximum reductions compared to the soybean-based diet were estimated 4%, 7% and 9% for GWP_100_, EP and AP, respectively.

The present study focused on the environmental impacts of a broiler production system for three diets that have not been examined in the literature. Its results are in the same order of magnitude with the results reported in relevant recent studies. Prudêncio da Silva *et al*. [23] reported the values 0.0287–0.0472 kg SO_2_eq for AP, 0.0138–0.0193 kg PO_4_^3-^eq for EP, 1.450–2.700 kg CO_2_eq for GWP_100_, 2.47–3.9 m^2^·year for ALO and 18–29.5 MJ for CED for the different systems that they examined. Also, Gonzalez-Garcia *et al*. [22] reported the values 0.0305 kg SO_2_eq, 0.0143 kg PO_4_^3-^eq, 1.588 kg CO_2_eq and 10.941 MJ for AP, EP, GWP_100_ and CED, respectively. The differences from this study could be attributed to differences in the systems studied and the LCA methodology followed (e.g. differences in broiler rations, emission factors, characterization factors etc.). Furthermore, the potential reductions in AP (3.7%), EP (1.7%) and GWP_100_ (6.9%) corresponding to the Soy-Prot system that were estimated in this study are close to those reported in the studies of Bundgaard *et al*. [25] (5–9% for GWP_100_) and Leinonen and Williams [26] (5–9% for AP, 3–7% for EP and 2–4% for GWP_100_,). These two studies examined broiler diet systems in which soybean meal were slightly decreased and digestibility increasing enzymes were added.

#### Environmental hot-spots and differences in the environmental performance

Combining the results presented in Tables 11 and 12 for GWP_100_ regarding the emissions of CO_2_ from direct land transformation and soybean production, respectively, it is revealed that soybean production is the dominant factor to drive CO_2_ emissions due to direct land transformation. This implies that the CO_2_ emission from direct land transformation during soybean production is a hot-spot regarding the GWP_100_ of the Control and Soy-Prot systems. The hot-spots for the rest of the EICIs and each system are presented in Table 13. The environmental hot-spots constitute the flows of the processes within the system boundaries whose manipulation should be a priority in order to improve the performance of the system regarding each EICI. Table 13 indicates that the majority of the environmental hot-spots for the three systems occur off-farm. Soybean production in Latin American countries, domestic corn production and domestic lignite mining are important polluting processes for the life cycles of Control and Soy-Prot systems, with hot-spots in 7, 6 and 3 EICIs, respectively. As expected, domestic corn production becomes more important for the Gluten-Prot system with hot-spots in 7 EICIs, while soybean production less important (hot-spot in 1 EICI). Furthermore, from the farmer’s perspective, the annual NH_3_ and N_2_O emissions due to litter management are important flows to deal with in order to mitigate the AP and EP and the GWP_100_ for the Control and Soy-Prot systems and the Gluten-Prot system, respectively. The alternative diets tested only slightly affect these potential NH_3_ and N_2_O emissions. This is explained by the fact that the estimation of these emissions is solely dependent on the CP intake which is in turn slightly altered between the diets tested (Tables 1 and 5). It should also be mentioned that the effect of the protease on the broilers’ nitrogen retention was not considered in this estimation. This is an issue which requires further experimental investigation.

**Table 13:** Hot-spots for the EICIs and each system studied.

EICI	System / Hot-spots		
	Control	Soy-Prot	Gluten-Prot
ADP	P use (raw material) in phosphate rock fertilizer production (WE^a^)	P use (raw material) in phosphate rock fertilizer production (WE^a^)	P use (raw material) in phosphate rock fertilizer production (WE^a^)
AP	NH_3_ (air) from broilers’ manure management (Gr^a^)	NH_3_ (air) from broilers’ manure management (Gr^a^)	NH_3_ (air) from broilers’ manure management (Gr^a^)
	NH_3_ (air) from soybean production (Br^a^, Ar^a^)	NH_3_ (air) from soybean production (Br^a^, Ar^a^)	NH_3_ (air) from corn production (Gr^a^)
	NH_3_ (air) from corn production (Gr^a^)	NH_3_ (air) from corn production (Gr^a^)	
EP	NO_3_^-^ (water) and NH_3_ (air) from soybean production (Br^a^, Ar^a^)	NO_3_^-^ (water) and NH_3_ (air) from corn production (Gr^a^)	NO_3_^-^ (water) and NH_3_ (air) from corn production (Gr^a^)
	NH_3_ (air) from broilers’ manure management (Gr^a^)	NO_3_^-^ (water) and NH_3_ (air) from soybean production (Br^a^, Ar^a^)	NH_3_ (air) from broilers’ manure management (Gr^a^)
	NO_3_^-^ (water) and NH_3_ (air) from corn production (Gr^a^)	NH_3_ (air) from broilers’ manure management (Gr^a^)	PO_4_^3-^ (water) from lignite mining (Gr^a^)
	PO_4_^3-^ (water) from lignite mining (Gr^a^)	PO_4_^3-^ (water) from lignite mining (Gr^a^)	
POP	NO_x_ (air) from diesel in machinery(All^a^)	NO_x_ (air) from diesel in machinery (All^a^)	NO_x_ (air) from diesel in machinery (All^a^)
	NO_x_ (air) from transport, truck (<10t) (Gr^a^)	NO_x_ (air) from transport, truck (<10t) (Gr^a^)	NO_x_ (air) from transport, truck (<10t) (Gr^a^)
	NO_x_ (air) from transport sea ship, (80000 DWT) (All^a^)	NO_x_ (air) from transport sea ship, (80000 DWT) (All^a^)	
ALO	Arable land (raw material) for soybean production (Ar^a^, Br^a^)	Arable land (raw material) for soybean production (Ar^a^, Br^a^)	Arable land (raw material) for corn production (Gr^a^)
	Arable land (raw material) for corn production (Gr^a^)	Arable land (raw material) for corn production (Gr^a^)	Arable land (raw material) for wheat production (Gr^a^)
NLT	Transformation from forest (raw material) in soybean production (Ar^a^, Br^a^)	Transformation from forest (raw material) in soybean production (Ar^a^, Br^a^)	Transformation from forest (raw material) in soybean production (Ar^a^, Br^a^)
CED	Crude oil (raw material) for diesel production (All^a^)	Crude oil (raw material) for diesel production (All^a^)	Crude oil (raw material) for diesel production (All^a^)
	Lignite (raw material) for lignite mining (Gr^a^)	Lignite (raw material) for lignite mining (Gr^a^)	Lignite (raw material) for lignite mining (Gr^a^)
			Crude oil (raw material) for heavy fuel oil (Gr^a^)
GWP_100_	CO_2_ (air) due to land transformation in soybean production (Ar^a^, Br^a^)	CO_2_ (air) due to land transformation in soybean production (Ar^a^, Br^a^)	CO_2_ (air) due to fossil fuel combustion in high voltage electricity production from lignite (Gr^a^)
			N_2_O (air) from corn production (Gr^a^)
			N_2_O (air) from broilers’ manure management (Gr^a^)
HTPc	Chromium VI (Cr(VI)) (water) from lignite mining (Gr^a^)	Chromium VI (Cr(VI)) (water) from lignite mining (Gr^a^)	Chromium VI (Cr(VI)) (water) from lignite mining (Gr^a^)
HTPnc	Zinc (Zn) (soil) from soybean production (Br^a^, Ar^a^)	Zinc (Zn) (soil) from soybean production (Br^a^. Ar^a^)	Zinc (Zn) (soil) from corn production (Gr^a^)
	Zinc (Zn) (soil) from corn production (Gr^a^)	Zinc (Zn) (soil) from corn production (Gr^a^)	Zinc (Zn) (soil) from wheat production (Gr^a^)
FWETP	Chlorpyrifos (soil) from soybean production (Ar^a^)	Zinc (Zn) (water) from lignite mining (Gr^a^)	Zinc (Zn) (water) from lignite mining (Gr^a^)
	Zinc (Zn) (water) from lignite mining (Gr^a^)	Chlorpyrifos (soil) from soybean production (Ar^a^)	Alachlor (soil) from corn production (Gr^a^)
	Zinc (Zn) (soil) from soybean production (Br^a^, Ar^a^)	Zinc (Zn) (soil) from soybean production (Br^a^, Ar^a^)	Zinc (Zn) (water) from lignite ash treatment (Gr^a^)
	Zinc (Zn) (soil) from corn production (Gr^a^)	Zinc (Zn) (soil) from corn production (Gr^a^)	
WDI	Water use in corn production (Gr^a^)	Water use in corn production (Gr^a^)	Water use in corn production (Gr^a^)
	Water use in wheat production (Gr^a^)	Water use in wheat production (Gr^a^)	Water use in wheat production (Gr^a^)

^a^ The origin is specified. All: all production locations and imports by ship to Greece, Gr: Greece, Ar: Argentina, Br: Brazil, WE: Western Europe

Small differences in all EICIs between the Control and the Soy-Prot systems were observed in this study (Table 10). They could be attributed to the 5.5% increase in corn use as well as to the 9% and 17% decrease in SBM and soybean oil use in the broilers’ diet between the two systems (Table 1). These modifications, for the studied partial life cycle, result in (Tables 11 and 12) lower use of phosphate rock fertilizer (ADP), less NO_x_ emissions due to fuel combustion in agricultural operations and transports (POP), smaller occupation of agricultural land (ALO), lower diesel use for agricultural operations (CED), less emissions to soil due to the use of fertilizers and pesticides in crop production processes (HTPnc), lower amount of total ammonia emissions (AP and EP) and modifications in soil and water pollution causing freshwater ecotoxocity. Moreover, the increase in corn use leads to higher water consumption (higher WDI in the ‘Soy-Prot’ system) as well as higher domestic electricity consumption (increased lignite mining operation) for corn’s drying, affecting the EP, the CED and the FWETP (Tables 11 and 12). This higher electricity demand also triggers an increase in HTPc in the Soy-Prot system compared to the Control system (Table 12). Furthermore, a reduction of ammonia emissions mainly from the foreground system (i.e. from the on-farm litter management), the soybean production and the corn production, largely contributed to the potential reduction of AP and EP values of the Soy-Prot system (Tables 11 and 12). The decreased soybean supply is associated with a reduction in the direct Land Use Change (LUC) from forests to arable soybean production land, resulting in less NLT and less CO_2_ emissions (Tables 11 and 12).

The complete substitution of soybean-based constituents in the broilers’ diet (Table 1) is the main reason for the potentially high reductions that occurred in the ADP, POP, ALO, NLT, GWP_100_ and HTPnc values between the Gluten-Prot and the Control systems (Table 10). For these indicators, the same justification as presented in the previous paragraph can be adopted. Furthermore, the 9% increase in corn supply to broilers (Table 1), in combination with the fact that the foreground system’s contribution remains almost on the same level (due to the combination of the level of ammonia decrease and the level of broilers’ growth decrease) (Tables 5 and 10), yields a decrease in the AP, counteracting the complete substitution of soybean production. The aforementioned factors along with the increase in domestic electricity requirements (to dry the corn and the wheat and to produce CGM and wheat bran) offset the effect of soybean production and trigger a decrease of EP. Moreover, the combined effect of this electricity utilization and the use of heavy fuel oil for steam production in the domestic Feed Industry results in an increase of CED (Table 12). In addition, the increase in the HTPc value is a direct consequence of the increased domestic electricity consumption (Table 12). The increased corn and wheat use is related to the increased water demand when cultivating these crops, explaining the potential increase in the WDI value (Table 12). Furthermore, the decrease of FWETP is attributed to the increased corn production and to the increased electricity consumption (Table 12), compensating again for the effect of soybean production.

## Conclusions

Reducing the protein concentration in the diets for broilers with the addition of protease enzyme or the substitution of SBM by CGM together with the use of a protease could be potential dietary strategies to lower feeding cost and improve the environmental impact of farming. This study showed that the group fed less SBM protein with the addition of protease, showed satisfactory performance and gut health characteristics. Also, the substitution of SBM by CGM together with a protease marginally retained growth performance parameters, affected positively the intestinal microflora of broiler chickens and retained gut integrity. Although the substitution of SBM by CGM marginally increased the feed cost, the overall feed cost per weight gain did not differ among the three groups. The reduction of SBM in feed inclusion at lower level along with protease or substitution of SBM by CGM and protease did not affect the chemical composition of broiler meat. In the current study, it was shown that it is possible to reduce SBM and feed crude protein content by the addition of protease enzyme. It was also shown that the substitution of SBM by CGM together with a protease enzyme is economically viable. However, further research is needed to investigate the relation of feed digestion and growth performance of broilers challenged with bacteria or protozoa that cause severe intestinal diseases.

The LCA performed indicated that the N related emissions (NH_3_ and N_2_O) due to litter handling are the most important farm-level flows to be dealt with in order to reduce the acidification, eutrophication and global warming effects caused by the studied partial life-cycles. However, the number of the off-farm environmental hot-spots suggests that interventions in different parts of the production chain (especially in Latin American soybean production and domestic corn production and lignite mining processes) would be required for improvement of its environmental performance. This analysis further showed a potential decrease for the majority of the EICIs for the Gluten-Prot system in comparison to the conventional Control system. For most of these EICIs, this decrease was larger in the Gluten-Prot system than in the Soy-Prot system. Both the complete substitution of SBM and soybean oil and the increase in corn use (both for corn grain and CGM consumption) are regarded as the main cause of these reductions. This study finally suggests that the Gluten-Prot diet would provide an eco-friendly alternative to the Control diet of the specific system, if the water depletion during domestic corn and wheat production reduced and if electricity production in Greece became less dependent on lignite. In this study, LCA was used to provide deterministic values for the environmental impacts of the systems defined by the different diets tested which are regarded as initial, indicative figures on their environmental performance. Therefore, future LCA research should be focused on detecting and handling uncertainty issues regarding the environmental impacts of Greek broiler production processes in order to statistically support comparisons between the systems defined for different broiler diets.

The maintenance of the broilers’ growth performance (body weight, feed intake and FCR), meat quality, feed cost per kg weight gain and gut integrity, the positive effect on the intestinal microflora and the potential reduction in the majority of the environmental impacts, could all be considered as positive indications in the effort to sustainably replace the conventional, soybean-dependent Control diet in the specific broiler production system. More research is required in order to identify alternative broiler diets with positive effects on broilers’ health and growth characteristics, the system’s economic performance, the broiler meat quality characteristics and all the EICIs, thus towards more sustainable Greek broiler production systems.

## Supporting Information

S1 Table Data set for broiler chickens’ performance parameters. (XLSX)

S2 Table Data set for LCA analysis. (XLSX)

## Abbreviations

ADP: Abiotic Depletion Potential
ALO: Agricultural Land Occupation
AP: Acidification Potential
ASAE: American Society of Agricultural Engineers
CD: Crypt Depth
CED: Cumulative Energy Demand
CGM: Corn Gluten Meal
CP: Crude Protein
CTUe: Comparative Toxic Units (Ecological)
CTUh: Comparative Toxic Units (Human)
DM: Dry Matter
EDTA: Ethylenediaminetetraacetic acid
EICI: Environmental Impact Category Indicator
ELCD: European Life Cycle Database
EMEP/EEA: European Monitoring and Evaluation Programme / European Environment Agency
EP: Eutrophication Potential
FAO: Food and Agriculture Organization of the United Nations
FCR: Feed Conversion Ratio
FU: Functional Unit
FWETP: Freshwater Ecotoxicity Potential
GMO: Genetically Modified
GWP_100_: Global Warming Potential (100 years framework)
HTPc: Human Toxicity Potential (cancer effects)
HTPnc: Human Toxicity Potential (non-cancer effects)
IPCC: Intergovernmental Panel of Climate Change
LCA: Life Cycle Assessment
LCI: Life Cycle Inventory
LCIA: Life Cycle Impact Assessment
LEAP: Livestock Environmental Assessment and Performance Partnership
LW: Live Weight
NLT: Natural Land Transformation
NMVOC: Non Methane Volatile Organic Compounds
PBS: Phosphate Buffered Saline
PCR: Polymerase Chain Reaction
POP: Photochemical Ozone Formation Potential
PRA: Protease RONOZYME^®^ Proact
SBM: Soybean Meal
VH: Villous Height
WDI: Water Depletion Index
